# Moxibustion Inhibits the Expression of Colonic NLRP3 through miR7/RNF183/NF-*κ*B Signaling Pathway in UC Rats

**DOI:** 10.1155/2021/6519063

**Published:** 2021-11-03

**Authors:** Xi-Ying Li, Yan-Ting Yang, Yue Zhao, Xie-He Kong, Guang Yang, Jue Hong, Dan Zhang, Xiao-Peng Ma

**Affiliations:** ^1^Shanghai Municipal Hospital of Traditional Chinese Medicine, Shanghai University of Traditional Chinese Medicine, Jingan District, Shanghai 200071, China; ^2^Key Laboratory of Acupuncture-Moxibustion and Immunology, Shanghai Research Institute of Acupuncture and Meridian, Xuhui District, Shanghai 200030, China

## Abstract

**Background:**

Moxibustion has been recognized as an effective approach for ulcerative colitis, yet its mechanism is not clear. The research aimed to investigate the influence of moxibustion on the activation of NLRP3 inflammasome and its mechanism in treating ulcerative colitis by observing miR7/RNF183 inducing I*κ*B *α* ubiquitination to regulate NF-*κ*B signaling pathway in an ulcerative colitis rat model.

**Methods:**

An ulcerative colitis rat model was established by unlimited access to self-administration of 3.5% (w/v) dextran sulfate sodium solution. Mild moxibustion was applied to bilateral Tianshu points (ST25) in the moxa-stick moxibustion group; rats in the control group were intervened by intraperitoneal injection of ubiquitination inhibitor, MG132. The disease activity index was determined at the end of the intervention; colon injury was observed and scored after hematoxylin-eosin staining; the immunohistochemical method was adopted to detect the expressions of colonic IL-1*β* and NLRP3 proteins; Western blot determined the expressions of RNF183, I*κ*B *α*, and NF-*κ*B p65 proteins in the colon; the immunofluorescence test was used to observe the coexpression of I*κ*B *α*/ubiquitin and I*κ*B *α*/RNF183 proteins in the colon; immunoprecipitation assay was adopted to observe the interaction between I*κ*B *α* and RNF183 proteins; and quantitative real-time polymerase chain reaction determined the expression of colonic miR7.

**Results:**

Moxibustion lowered the disease activity index, manifesting as restored colonic tissue and reduced inflammatory reaction, and decreased expression levels of NLRP3 and IL-1*β* proteins, compared with the model group. It also reduced colonic expression of NF-*κ*B p65 protein, together with the increased level of I*κ*B *α* protein and weaker expression levels of ubiquitin and RNF183 proteins and mRNAs and stronger expression of miR7. There were no significant differences between the moxa-stick moxibustion group and the control group except the expressions of RNF183 protein and mRNA and miR7.

**Conclusion:**

Moxibustion encourages the recovery of colon injury probably by regulating the expression of NLRP3 protein in ulcerative colitis rats through miR7/RNF183/NF-*κ*B signaling pathway.

## 1. Introduction

Ulcerative colitis (UC) is an idiopathic chronic inflammatory bowel disease (IBD), which mainly attacks the rectum, colonic mucosa, and submucosa [1], with diarrhea, mucous stool, bloody stool, tenesmus, and cramping abdominal pain as its clinical symptoms [2]. UC is prevalent and intractable [3], with an incidence of around 0.5 million cases per year, and younger population between ages 17 and 40 years are more common [2]. The incidence in China has been rising with each passing year [4]. Due to its complexity and recurrence, patients' quality of life (QOL) is greatly impacted, and the prognosis is unpleasant [5]. Therefore, prevention of UC, induction of clinical remission, and slowing down its progression are of great significance.

When NOD-like receptor protein 3 (NLRP3) inflammasome is overactivated, it will release the downstream inflammatory factor interleukin (IL)-1*β*, which plays a key role in damaging the colonic barrier and causing inflammatory injuries. Hence, modulation of NLRP3 inflammasome activation is beneficial to the inhibition of inflammation and the reconstruction of colonic mucosal immune homeostasis [6]. Ubiquitination modification has been proved to be one of the effective ways to regulate NLRP3. The ubiquitinated cascade-related proteins in the nuclear factor kappa B (NF-*κ*B) signaling pathway participate in the inflammatory response of IBD via regulating the transcription of NLRP3 and its downstream inflammatory factors [7]. Ring finger protein 183 (RNF183) promotes the degradation of ubiquitinated I*κ*B *α* and boosts the nuclear translocation of NF-*κ*B p65, triggering intestinal inflammation [8]. Therefore, it may be one part of the mechanism to suppress the activation of NLRP3 inflammasome and alleviate colonic inflammatory injury in UC through regulating the ubiquitination modification in the NF-*κ*B signaling pathway.

In recent years, there has been a surge in the clinical reports of moxibustion treatment of IBD [9–11], which all showed satisfactory improvements in symptoms like abdominal pain and diarrhea, and thus, moxibustion has been recommended as an adjuvant treatment in clinical practice [12]. However, it still remains a mystery how this treatment works. Our previous findings manifested that moxibustion downregulated the expression levels of NLRP3, IL-1*β*, and ubiquitin (Ub) proteins in the colon of UC rats so as to fulfill its anti-inflammatory function and repair inflammatory damage in the colon [13]. It requires in-depth studies to find out if RNF183 is involved for moxibustion to regulate the ubiquitination level of the NF-*κ*B signaling pathway in colon tissue and inhibit the expression of NLRP3. The current study adopted a rat UC model established using dextran sulfate sodium (DSS) to observe the relationship between the RNF183-induced I*κ*B *α* ubiquitination and the expression of NLRP3 in colon tissue and the regulatory effect of moxibustion, for elaborating the anti-inflammatory immune regulation of moxibustion in the treatment of UC from the perspective of the signal pathway.

## 2. Materials and Methods

### 2.1. Animals

Forty-four clean male Sprague–Dawley (SD) rats weighing 150 ± 10 g were purchased from Shanghai SLAC Laboratory Animal Co. Ltd. (SYXK(hu)2014–0008) and were bred in the Laboratory Animal Center of Shanghai University of Traditional Chinese Medicine. After three-day adaptive feeding and exclusion of unhealthy rats by observing their diet, activity, posture, hair, etc., the experiment started. All experimental operations strictly followed the Guiding Opinions on the Treatment of Experimental Animals (National Science-Technology and Finance (2006).398). This experiment has been approved by the Animal Ethics Committee of Shanghai University of Traditional Chinese Medicine (PZSHUTCM18111612).

### 2.2. Reagents and Instruments

Reagents and instruments used in the study are as follows: DSS (MP Biomedicals, UK); moxa sticks for animal experiments (4 mm in diameter and 12 cm in length, Nanyang Hanyi Moxa Co. Ltd., China); ubiquitination inhibitor MG132 (Sigma, USA); NLRP3, IL-1*β* and Ub primary antibodies (Abcam, UK); RNF183 primary antibody (Novus, USA); I*κ*B *α* and NF-*κ*B p65 primary antibodies (CST, USA); fluorescent secondary antibodies (Alexa Fluor 647 and Alexa Fluor 488, Abcam, UK); anti-fluorescence quenching sealing liquid (containing DAPI, Beyotime Biotechnology Co. Ltd., China); immunoprecipitation (IP) kit (Thermo Fisher, USA); immunohistochemical detection kit (Dako, Denmark); immunohistochemical detection instruments for dehydration, paraffin-embedding, baking, and spreading (Leica, Germany); fluorescence microscope (Olympus, Japan); Western blotting (WB) instruments for electrophoresis, wet transfer, and gel imaging system (Bio-Rad, USA); and real-time fluorescent quantitative PCR equipment (Roche, Switzerland).

### 2.3. UC Rat Modeling

The UC rat model was prepared by unlimited access to self-administration of 3.5% (w/v) DSS solution (molecular mass 36,000–50,000) for 7 consecutive days [14]. At the end of modeling, one rat was randomly selected from each group for model identification. Intervention would begin given the success of the model.

### 2.4. Intervention

**Figure 1:**
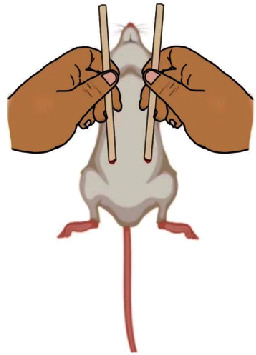
Diagram of moxibustion.

The 44 rats were randomly divided into four groups: normal group (NG), model group (MG), moxa-stick moxibustion group (MSMG), and control group (CG), 11 each. Rats in the MSMG were given moxibustion at bilateral Tianshu (ST25) points [15], that is, a moxa stick was ignited and suspended over the acupoints by 2–3 cm away, 10 min each time, once a day, for a total of 8 days (Figure 1). Rats in the CG received intraperitoneal injection of ubiquitination inhibitor MG132, 2.1 mL/kg each time, once a day for 8 consecutive days [16]. Rats in the MG did not receive any intervention but the same grasping and fixing as in the MSMG. Rats in the NG were not subjected to modeling or treatment but only grasped and fixed in the same way as in the MSMG. During treatment, all the rats were provided with free access to 1% DSS solution to maintain the model stability except for those in the NG.

### 2.5. Disease Activity Index (DAI)

According to the DAI scoring standard [17, 18], the rats were observed and scored. DAI = (weight loss rate score ＋ stool form score ＋ fecal occult blood score)/3; weight loss rate (%) = (weight before experiment − weight after modeling)/weight before experiment × 100%.

### 2.6. Colonic Morphology

Hematoxylin-eosin (HE) staining was used to observe and score the changes in the colonic morphology under an optical microscope [19].

### 2.7. Immunohistochemistry (IHC)

After the colon tissue sections were deparaffinized, hydrated, antigen retrieved, and sealed, the primary antibody (NLRP3 1 : 300; IL-1*β* 1 : 50) was added dropwise and incubated overnight at 4°C. On the next day, the secondary antibody (1 : 200) was added dropwise and incubated at room temperature for 1 h; DAB developed. After hematoxylin-counterstained for 1 min, the sections were differentiated, dehydrated, transparentized, and mounted. The tan particles found in the cytoplasm were taken as the positive target. Five visual fields were randomly selected from each slice to be photographed, and the Image-Plus Pro 6.0 software was used to analyze and calculate the integral optical density (IOD) of each photo. The average value was taken as the IOD of the slice.

### 2.8. Western Blotting (WB)

RIPA lysis buffer was added to colon tissues to extract the total protein, and protein quantification was performed by the BCA method. The proteins were separated by SDS-PAGE gel electrophoresis and transferred to the PVDF membrane. Then, the PVDF membrane was blocked with 5% BSA for 1 h, and the primary antibody (RNF183 1 : 1000; I*κ*B *α* 1 : 1000; NF-*κ*Bp65 1 : 1000) was added and incubated overnight at 4°C. On the next day, HRP^+^ secondary antibody (1 : 1000) was added dropwise and incubated at room temperature for 1 h, and the expression of the target protein was detected by the ECL chemiluminescence method. The PVDF membrane was reblocked for 1 h; HRP + GAPDH primary antibody (1 : 1000) was added and incubated for 1 h, and the expression of internal reference protein was detected. The Image J software was adopted to analyze the gray value of the corresponding band and calculate the ratio of the target protein to the internal reference protein.

### 2.9. Real-Time Fluorescent Quantitative PCR (RT-qPCR)

Trizol was added to the colon tissue to extract the total RNA. PCR amplification was carried out after the synthesis of the first cDNA. The procedure was as follows: 95°C for 10 min; (95°C 15 s; 60°C 45 s) × 40 cycles. The data were collected and analyzed by ABI Prism 7300 SDS software, and the obtained data were converted into 2△Ct(△Ct = target gene Ct value-reference GAPDH Ct value) to obtain the relative expression of the target gene mRNA. Primer sequence is as follows: RNF183: 5′ CGTCACCCTGCTTCTCATC 3′, 5′ CTGTCGGGCATCTGTTCTC 3′, 219 bps; GAPDH: 5′ GGAGTCTACTGGCGTCTTCAC 3′, 5′ ATGAGCCCTTCCACGATGC 3′, 237 bps; miR-7: 5′ GCGCGTGGAAGACTAGTGATTT 3′, 5′ AGTGCAGGGTCCGAGGTATT 3′, 79 bps; U6: 5′ CTAAAATTGGAACGATACAG 3′, 5′ AAATATGGAACGCTTCAC 3′, 81 bps.

### 2.10. Immunofluorescence (IF) and Colocalization

After deparaffinization, hydration, antigen retrieval, and sealing of the colon tissue sections, the primary antibody (Ub 1:500) was added and incubated overnight at 4°C. On the next day, a fluorescent secondary antibody (1 : 200) was added in shade and incubated at 37°C for 45 min, counterstained with DAPI counterstain for 10 min, and then mounted. One visual field was chosen and activated with excitation light of the corresponding wavelength. DAPI-stained nucleus showed blue, and the positive expression of Ub protein was in red. Pictures were taken and merged. Five fields were randomly selected from each slice; the IOD of the positive target was calculated; and the average was taken for analysis.

Colocalization of different proteins is as follows: the first primary antibody (I*κ*B *α* 1 : 200) was treated as above. Before counterstaining with DAPI counterstain, 5% BSA was used for reblocking and incubated for 1 h at room temperature. The second primary antibody (Ub 1 : 500; RNF183 1 : 200) was then added and incubated at 37°C for 2 h. Afterwards, the second fluorescent secondary antibody (1 : 200) was added and incubated at 37°C for 1 h. Finally, the nucleus was counterstained with DAPI for 10 min and then mounted. A field was chosen and activated with excitation light of the corresponding wavelength. DAPI-stained nucleus was in blue; positive Ub and RNF183 protein expression were in red; and positive I*κ*B*α* protein was in green. Pictures were taken and merged.

### 2.11. Immunoprecipitation (IP)

IP protein lysate was added to the colon tissues for extracting the total protein, and the protein concentration was determined by the BCA method. The cross-linking buffer and primary antibody were added into a spin column containing the resin, and they were incubated at room temperature to bind the antibody to protein A/G agarose. Then, cross-linking buffer and DSS (disuccinimidyl suberate) were added to the spin column for cross-linking the combined antibody. Antigen immunoprecipitation was completed by adding the pretreated tissue lysate into the spin column containing the primary antibody and cross-linked resin and incubated at 4°C overnight. After the spin column was added the elution buffer, incubated, and centrifuged, the antigen eluent was obtained. And, the loading buffer was added into the eluted protein solution for SDS-PAGE analysis. Finally, Ub or RNF183 primary antibody was added for incubation, and the expression of target protein in elution protein solution was detected by WB in order to observe the ubiquitination of I*κ*B *α* and interaction of RNF183-I*κ*B *α*.

### 2.12. Statistical Analysis

All data were statistically processed using SPSS version 25.0 statistical software. The measurement data were firstly tested for distribution. Those conforming to normal distribution were expressed as $\bar{X}\pm S$, while those not were expressed as median (*P*_25_, *P*_75_). Between-group differences were analyzed using one-way ANOVA when it was normal distribution and homogenous variance, and the least significant difference (LSD) test for pairwise comparisons. For heterogeneous variance, the Games–Howell test was applied. A nonparametric test was employed for data of skewed distribution. The test level was set as *α* = 0.05, and *P* < 0.05 was recognized as statistical significance.

## 3. Results

### 3.1. DAI

**Figure 2:**
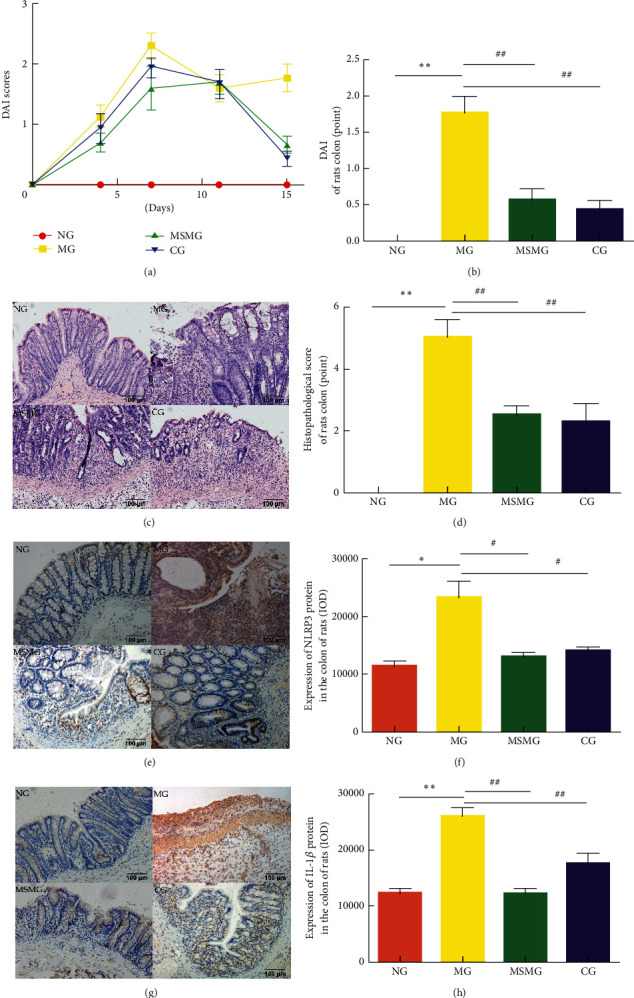
Comparison of DAI, colonic morphology, pathological damage score, and the expression of IL-1*β* and NLRP3 proteins in colon: (a) change in DAI in each group; (b) comparison of DAI in the end of experiment; (c) colonic morphology under the microscope by HE staining (amplification × 200); (d) colonic pathological damage score in each group; (e) immunohistochemical staining of IL-1*β* protein in rat's colon tissue in each group (amplification × 200); (f) IOD of IL-1*β* in each group; (g) immunohistochemical staining of NLRP3 protein in rat's colon tissue in each group (amplification × 200); and (h) IOD of NLRP3 in each group. NG: normal group; MG: model group; MSMG: moxa-stick moxibustion group; CG: control group; DAI: disease activity index; IOD: integral optical density. Data expressed as mean ± SEM, *n* = 10 per group; vs. NG, ^*∗*^*P* < 0.05 and ^*∗∗*^*P* < 0.01; vs. MG, ^#^*P* < 0.05 and ^##^*P* < 0.01; and vs. MSMG, ^Δ^*P* < 0.05 and ^ΔΔ^*P* < 0.01.

Before treatment, the DAI score was significantly higher in the MG, MSMG, and CG compared with the NG (all *P* < 0.01). After treatment, the DAI score was significantly higher in the MG than in the NG (*P* < 0.01); compared with the MG, the DAI score dropped notably in the MSMG and CG (both *P* < 0.01); there was no significant difference between the CG and MSMG (*P* > 0.05; Figures 2(a) and 2(b)).

### 3.2. Morphological Observation of the Colon and Pathological Damage Score

Under the microscope, the colonic mucosal epithelium of rats in the NG was rather complete, with regularly arranged well-formed glands, and the submucosal connective tissues were not infiltrated by inflammatory cells, congested, or swollen. However, rats in the MG had severe colon injury, demonstrating incomplete tissue structure, mucosal epithelium, and gland defects, continuous ulcer formation, some crypt abscesses, notable swelling in connective tissues, and significant inflammatory cell infiltration. Compared with the NG, the colonic pathology score was significantly higher in the MG (*P* < 0.01). In the MSMG and CG, the structure of colonic mucosa was substantially intact, with new epithelial cells and gland hyperplasia, ulcers basically healed, and the submucosal connective tissues had mild inflammatory cell infiltration. Compared with the MG, the score of colonic pathological damage was significantly reduced in both MSMG and CG (both *P* < 0.01; Figures 2(c) and 2(d)).

### 3.3. Expression of NLRP3 and IL-1*β* Proteins in the Colon

NLRP3 and IL-1*β* proteins were mainly expressed in intestinal mucosal epithelial cells, lymphocytes in the mucosal stroma, and monocyte/macrophage cytoplasm. Compared with the NG, the expression of colonic NLRP3 and IL-1*β* proteins increased significantly in the MG (*P* < 0.05 and *P* < 0.01, respectively). Compared with the MG, the expression of colonic NLRP3 and IL-1*β* proteins showed a notable decrease in both MSMG and CG (*P* < 0.05 and *P* < 0.01, respectively). There were no significant differences in the expression of colonic NLRP3 and IL-1*β* proteins between CG and the MSMG (both *P* > 0.05; Figures 2(e)–2(h)).

### 3.4. Expression of I*κ*B *α*, Ubiquitinated I*κ*B *α*, and NF-κB p65 Proteins in the Colon

**Figure 3:**
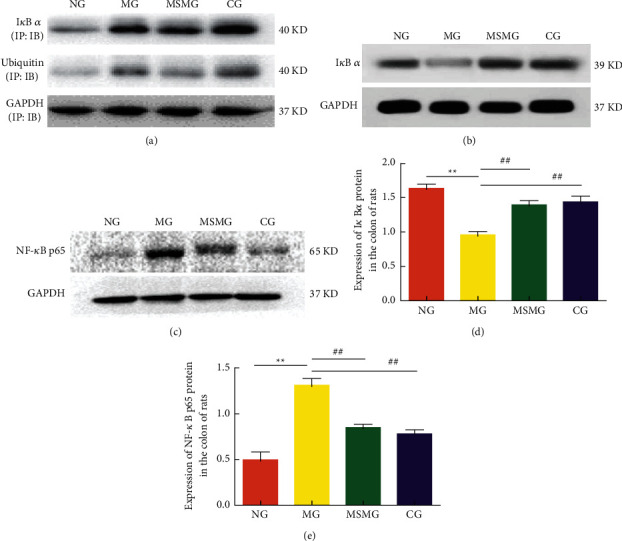
Ubiquitination of I*κ*B *α* and comparison of the expression of NF-*κ*B p65 and I*κ*B *α* proteins in colon: (a) the expression of ubiquitin and I*κ*B *α* proteins in each group after the immunoprecipitation (IP) via immunoblotting (IB); (b) representative Western blot result showing the expression of I*κ*B *α* protein in each group; (c) representative Western blot result showing the expression of NF-*κ*B p65 protein in each group; (d) quantification showing the expression of I*κ*B *α* protein in each group; and (e) quantification showing the expression of NF-*κ*B p65 protein in each group. NG: normal group; MG: model group; MSMG: moxa-stick moxibustion group; CG: control group. Data expressed as mean ± SEM, *n* = 10 per group; vs. NG, ^*∗*^*P* < 0.05 and ^*∗∗*^*P* < 0.01; vs. MG, ^#^*P* < 0.05 and ^##^*P* < 0.01; and vs. MSMG, ^Δ^*P* < 0.05 and ^ΔΔ^*P* < 0.01.

The colonic I*κ*B *α* protein was separated by IP, and then the eluent was incubated with the ubiquitin primary antibody for WB verification. Compared with the NG, the expression of ubiquitin in IP eluent had strengthened when the expression of I*κ*B *α* protein was downregulated in the MG (*P* < 0.01). Compared with the MG, the expression of ubiquitin in IP eluents weakened, and the expression of I*κ*B *α* protein in the colon of rats in the MSMG and CG was upregulated (all *P* < 0.01; Figures 3(a), 3(b), and 3(d)). Compared with the NG, the expression of colonic NF-*κ*B p65 protein was significantly upregulated in the MG (*P* < 0.01). Compared with the MG, the expressions of NF-*κ*B p65 protein decreased in the MSMG and CG (both *P* < 0.01). The differences in the expression of colonic NF-*κ*B p65 and I*κ*B *α* proteins were statistically insignificant between the CG and the MSMG (both *P* > 0.05; Figures 3(c) and 3(e)).

### 3.5. Expression of Ub Protein and Its Colocalization with I*κ*B *α*

**Figure 4:**
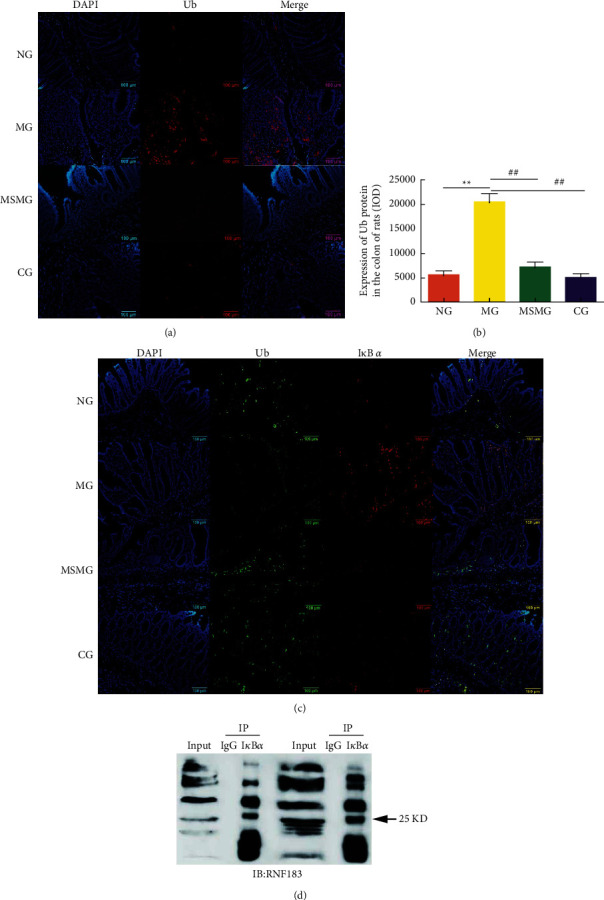
Expression of colonic Ub protein and colocalized expression of Ub and I*κ*B *α* proteins: (a) immunofluorescence staining of Ub protein in rat's colon tissue in each group (amplification × 200); (b) comparison of the Ub protein expression in rat's colon; and (c) immunofluorescence staining of colocalized expression of Ub and I*κ*B *α* proteins (amplification × 200). NG: normal group; MG: model group; MSMG: moxa-stick moxibustion group; CG: control group. Data expressed as mean ± SEM, *n* = 10 per group; vs. NG, ^∗^*P* < 0.05 and  ^*∗∗*^*P* < 0.01; vs. MG, ^#^*P* < 0.05 and ^##^*P* < 0.01; and vs. MSMG, ^Δ^*P* < 0.05 and ^ΔΔ^*P* < 0.01.

Ub protein was mainly distributed in the intestinal lamina propria and weakly expressed in the mucosal epithelium (Figure 4(a)). Compared with the NG, the expression of Ub protein in the colon was significantly stronger in the MG (*P* < 0.01); compared with the MG, the expression of Ub protein in colon tissue was notably weaker in the MSMG and the CG (both *P* < 0.01). There was no significant difference in the expression of Ub protein in the colon between the MSMG and CG (*P* > 0.05; Figures 4(a) and 4(b)). The immunofluorescence colocalization results revealed positive expression of Ub and I*κ*B *α* proteins in the mucosal epithelium, lamina propria, and colonic submucosa in each group. The positive expression of Ub protein was in green fluorescence, and the I*κ*B *α* protein was in red. The co-localized expression of the two proteins was in a color approaching yellow, mostly in the colonic mucosal lamina propria, which indirectly indicated their binding effect (Figure 4(c)).

### 3.6. Expression of RNF183 Protein and mRNA and Its Colocalization with I*κ*B *α*

**Figure 5:**
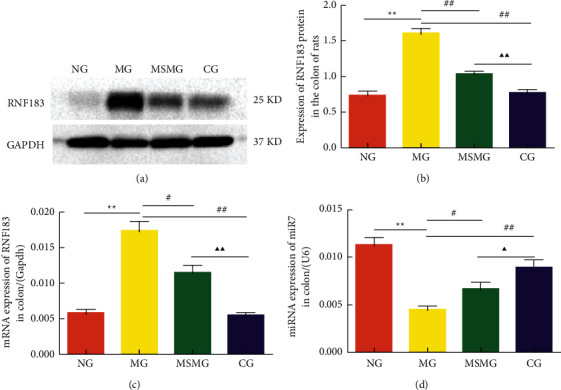
Comparison of the expression of colonic RNF183 protein and mRNA and miR7: (a) representative Western blot result showing the expression of RNF183 protein in each group; (b) quantification showing the expression of RNF183 protein in each group; (c) quantification showing the expression of RNF183 mRNA in each group; and (d) quantification showing the expression of miR7 in each group. NG: normal group; MG: model group; MSMG: moxa-stick moxibustion group; CG: control group. Data expressed as mean ± SEM, *n* = 10 per group; vs. NG, ^*∗*^*P* < 0.05 and ^*∗∗*^*P* < 0.01; and vs. MG, ^#^*P* < 0.05 and ^##^*P* < 0.01; and vs. MSMG, ^Δ^*P* < 0.05 and ^ΔΔ^*P* < 0.01.

**Figure 6:**
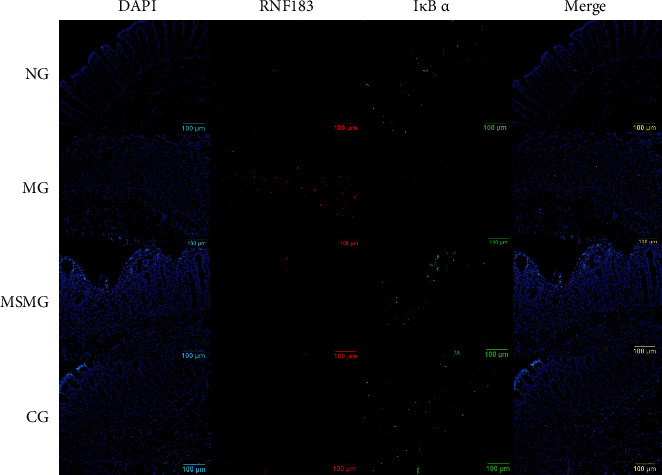
Colocalized expression of colonic RNF183 and I*κ*B *α* proteins. Immunofluorescence staining of RNF183 and I*κ*B *α* proteins in rat's colon tissue in each group (amplification × 200). NG: normal group; MG: model group; MSMG: moxa-stick moxibustion group; CG: control group.

Compared with the NG, the expression of colonic RNF183 protein and mRNA increased in the MG (both *P* < 0.01); compared with the MG, the expression of colonic RNF183 protein and mRNA reduced significantly in both the MSMG and the CG (all *P* < 0.05). There were also significant differences in the expression of RNF183 protein and mRNA in colon tissues between the MSMG and the CG (both *P* < 0.01; Figures 5(a)–5(c)). The immunofluorescence colocalization results showed positive expression of RNF183 and I*κ*B *α* proteins in the mucosal epithelium, lamina propria, and submucosa of the colon. The positive expression of RNF183 protein was in red fluorescence, and I*κ*B *α* protein was in green. The co-localized expression of the two was colored nearly yellow, more often found in the lamina propria (Figure 6). The results of IP probably further affirmed their binding effect (Figure 4(d)).

### 3.7. Expression of miR7 in the Colon

Compared with the NG, the expression of miR7 in the colon was significantly downregulated in the MG (*P* < 0.01); compared with the MG, the expression of miR7 in the colon was markedly upregulated in the MSMG and the CG (*P* < 0.05, *P* < 0.01). A significant difference was also found between the MSMG and the CG in comparing the expression of miR7 in colon tissue (*P* < 0.05; Figure 5(d)).

## 4. Discussion

UC is a common digestive disease that may severely affect the quality of life and mood of the patients due to long-term abdominal pain, diarrhea, bloody stools, and so on [20, 21]. Moxibustion therapy obtains its curative effect by thermal stimulation to acupoints generated by the burning of moxa. Known for its gentle action and safe operation, moxibustion works well in treating intestinal diseases mainly manifested by symptoms such as abdominal pain and diarrhea [22, 23]. In recent years, many studies have found that moxibustion can improve the clinical symptoms and mucosal damage in UC patients [24, 25], and it is an effective method worthy of attention and recommended in the treatment of UC [10]. An in-depth understanding of the mechanism of moxibustion in the treatment of UC will help provide a scientific basis for its clinical application. The current study demonstrated that moxibustion reduced DAI and the pathological score of the colon in UC rats, indicative of colonic mucosal repair and reduced inflammation, which is consistent with the previous research results [26, 27].

Although the pathogenesis of UC is still unclear, colon inflammation is recognized as the main pathological manifestation and underlying pathogenic mechanism, which makes anti-inflammation and promoting mucosal healing become the fundamental principles in UC treatment [28]. Under normal physiological conditions, NLRP3 inflammasome protects intestinal mucosa. However, under pathological conditions, an increased level of NLRP3 will cause inflammation. The study also showed that the inflammatory response induced by abnormally activated NLRP3 played an important role in the onset, progression, and prognosis of IBD [29]. Various pathogenic factors act on the intestinal mucosa in UC patients, inducing abnormal activation of NLRP3 inflammasome and elevating the expression of IL-1*β* and other inflammatory factors as well, subsequently causing topical intestinal mucosal damage. Knockout or inhibition of NLRP3 activation will help alleviate colon inflammation [30]. It is reported by a few studies that acupuncture can produce a certain regulatory effect on NLRP3 in a variety of diseases [31–33]. Our previous research also showed that moxibustion can inhibit the expression of colonic NLRP3 in IBD rats [13, 34]. In this study, we found that the expression of NLRP3 and IL-1*β* proteins in UC rat colon declined after moxibustion intervention, which may be part of the mechanism in anti-inflammation and colonic mucosa repair.

NLRP3 inflammasome is one of the most studied inflammasomes so far and has been found involved in many negative regulatory mechanisms [35]. Among them, NF-*κ*B is a crucial transcription factor that affects the expression of NLRP3 protein. The activation of NLRP3 inflammasome usually requires dual signals. The first signal, also the priming stage, is that the toll-like receptor (TLR) or tumor necrosis factor receptor (TNFR) induces the activation of NF-*κ*B, thereby upregulating the expression of NLRP3 and IL-1*β*/IL-18 precursor. There are two NF-*κ*B binding sites in the gene promoter region of NLRP3, which are necessary for NLRP3 gene transcription [36, 37], considered to be the key rate-limiting step in NLRP3 inflammasome activation. The results of this study showed that the expression of NF-*κ*B p65 protein was significantly upregulated in UC rat colon, accompanied by severe inflammatory damage to the colon; the abnormally increased NF-*κ*B p65 protein was reduced, and the colon damage was repaired after moxibustion, which were in line with the previous reports [38, 39]. Therefore, we can say that moxibustion may play its anti-inflammatory function by inhibiting the NF-*κ*B signaling pathway, reducing NLRP3 transcription, and suppressing NLRP3 activation.

In addition to phosphorylation, ubiquitination is another important way of protein modification that affects cell signaling pathway networks. Ubiquitination modification refers to the process of Ub covalently binding to the target protein under the catalysis of a series of enzymes. Its main function is protein degradation, clearance, and activity regulation. In IBD patients, mucosal M*φ* is the activated phenotype, and the expression of ubiquitin protease is significantly upregulated, manifested by antigen presentation and inflammatory response [40]. It can be seen that abnormal ubiquitination modification is involved in the onset and development of IBD, but the details are still under exploration. Research revealed that ubiquitination modification played an important role in regulating the activation of the NF-*κ*B signaling pathway [41], thus getting involved in the colonic inflammation [42]. E3 ligase is a key factor in ubiquitination modification. A variety of RING E3s, including RNF183, have been confirmed closely related to IBD [43]. Yu et al. [8] found that RNF183 can promote the degradation of ubiquitinated I*κ*B *α*, leading to nuclear translocation of NF-*κ*B p65, thereby inducing intestinal inflammation. At the same time, microRNA7 (miR7) can regulate RNF183 [44], so that inhibiting the activity of miR7 will help repair the TNBS-induced mucosal damage in IBD [45]. In this study, the expression of colonic miR7 increased; the expression of Ub and RNF183 proteins decreased; and the expression of I*κ*B *α* increased in the MSMG, together with notable improvement in colonic inflammation. It is suggested that moxibustion may inhibit the expression of RNF183 protein by regulating miR7, prevent the binding of I*κ*B *α* and RNF183, and carry out ubiquitination modification and degradation, thereby reducing the expression of NF-*κ*B p65, downregulating the transcription and activation of NLRP3, which may be one part of the mechanism in moxibustion treatment of UC.

However, there still exist some shortcomings in this study. Firstly, since we failed to find an appropriate RNF183 IP antibody, so we only did one-way IP in the co-IP test between RNF183 and I*κ*B *α* proteins in this experiment. Meanwhile, the immunofluorescence colocalization method showed certain interaction between the two proteins though the evidence was not sufficient. Therefore, based on the previous studies [8, 46] and the preliminary results of our experiment, we speculate that moxibustion can regulate I*κ*B *α* possibly through RNF183. Available RNF183 IP antibody, pull-down, mass spectrometry, and quantitative proteomics can help confirm the hypothesis and assess the effect of moxibustion, and the relevant experiments will be carried out in our future research. Secondly, the experiment used MG132 as the control for inhibiting ubiquitination. This drug can selectively inhibit proteasome, for example, it can inhibit the degradation of I*κ*B *α* mediated by the proteasome and effectively inhibit the activation of NF-*κ*B induced by TNF-*α* in A549 cells [47]. Hence, although this study suggested that MG132 may adjust the degradation of I*κ*B *α* by modulating miR7 and RNF183, it could also be a direct inhibition on the degradation, which may be one of the reasons for increased expression of I*κ*B *α* in the CG during the IP experiment. There is no evidence in this study but can only expect in vivo application of miR7 or RNF183 RNAi to confirm it, so as to provide a more comprehensive and reliable basis for further revealing the mechanism of moxibustion in treating UC.

## Data Availability

The research data used to support the findings of this study are included within the article.
